# Potential prognostic factors in progression-free survival for patients with cervical cancer

**DOI:** 10.1186/s12885-021-08243-3

**Published:** 2021-05-10

**Authors:** Hui-Hui Chen, Wei-Yu Meng, Run-Ze Li, Qing-Yi Wang, Yu-Wei Wang, Hu-Dan Pan, Pei-Yu Yan, Qi-Biao Wu, Liang Liu, Xiao-Jun Yao, Min Kang, Elaine Lai-Han Leung

**Affiliations:** 1 Zhuhai Hospital of Traditional Chinese and Western Medicine, Zhuhai City, Guangdong People’s Republic of China; 2 State Key Laboratory of Quality Research in Chinese Medicine/Macau Institute for Applied Research in Medicine and Health, Macau University of Science and Technology, Macau, People’s Republic of China

**Keywords:** Cervical cancer, Progression-free survival, Retrieved lymph nodes count, FIGO staging

## Abstract

**Background:**

Cervical cancer continues to be one of the leading causes of cancer deaths among females in low and middle-income countries. In this study, we aimed to assess the independent prognostic value of clinical and potential prognostic factors in progression-free survival (PFS) in cervical cancer.

**Methods:**

We conducted a retrospective study on 92 cervical cancer patients treated from 2017 to 2019 at the Zhuhai Hospital of Traditional Chinese and Western Medicine. Tumor characteristics, treatment options, progression-free survival and follow-up information were collected. Kaplan–Meier method was used to assess the PFS.

**Results:**

Results showed that the number of retrieved lymph nodes had a statistically significant effect on PFS of cervical cancer patients (*P* = 0.002). Kaplan-Meier survival curve analysis showed that cervical cancer patients with initial symptoms age 25–39 had worse survival prognoses (*P* = 0.020). And the using of uterine manipulator in laparoscopic treatment showed a better prognosis (*P* < 0.001). A novel discovery of our study was to verify the prognostic values of retrieved lymph nodes count combining with FIGO staging system, which had never been investigated in cervical cancer before. According to the Kaplan-Meier survival curve analysis and receiver operating characteristic (ROC) curve analysis, significant improvements were found after the combination of retrieved lymph nodes count and FIGO stage in predicting PFS for cervical cancer patients (*P* < 0.001, AUC = 0.826, 95% CI: 0.689–0.962).

**Conclusion:**

Number of retrieved lymph nodes, initial symptoms age, uterine manipulator, and retrieved lymph nodes count combining with FIGO staging system could be potential prognostic factors for cervical cancer patients.

**Supplementary Information:**

The online version contains supplementary material available at 10.1186/s12885-021-08243-3.

## Background

Globally, cervical cancer is the fourth of the most common cancers among females after breast, colorectal, and lung cancer [1]. In low and middle-income countries (LMICs), cervical cancer continues to be the second most prevalent cancers in morbidity and one of the leading causes of cancer deaths among females [2, 3]. It is a remarkable cause of mortality and morbidity among youthful and middle-aged women aged 20 to 39 years all over the world [4]. In January 2019, the National Central Cancer Registry of China (NCCRC) released its latest nationwide tumor statistics of population-based tumor registry data in 2015 gathered from 368 tumor registries in China. According to the number of female patients, cervical cancer ranks sixth in the incidence of malignant tumors in China. The overall crude incidence rate by Chinese standard population for cervical cancer was 16.56/100,000. Nearly 111,000 new cervical cancer cases were diagnosed in 2015, which alone has around 18.7% of the total cervical cancer in the world [3]. Due to the lack of valid prevention and screening methods, it is worth noting that morbidity of cervical cancer is still increasing in less-developed countries in recent years [5, 6]. Therefore, it appears to be an important step to find out potential prognostic factors in progression-free survival for patients with cervical cancer.

The prediction of clinical prognostic is the key factor in the therapeutic decision-making process. For cervical cancer, the therapeutic strategy generally depends on the clinical stage, which is established by the FIGO (International Federation of Gynecology and Obstetrics) staging system. The FIGO staging system exposes the extent and aggressiveness of cervical tumor, which is one of the known predictors for survivals. Nevertheless, as the FIGO staging was mainly based on clinical assessment of the anatomic extent, the error rate between the final histopathologic classification and the FIGO staging is about 25% in patients with early stages [7, 8]. Hence, it is inevitable to enhance the FIGO staging system for more precise and practical prognostication of cervical cancer patients, optimizing the life quality of long-term survival and individualized treatment.

Lymphadenectomy is the standard constituent part of cervical cancer patients treatment [9, 10]. Retrieved lymph nodes (RLN) count is considered as the way of objectively measure of the radicalness and thoroughness of the surgical procedure [11]. The range of lymphadenectomy is a matter of debate in the treatment of cervical cancer. Theoretically, lymphadenectomy could enhance the probability of detecting occult metastatic disease. In a study based on the single-institution review of cervical cancer patients, it has been reported that cervical cancer patients with more retrieved lymph nodes had better progression-free survival (PFS) [12]. And there is evidence investigated by other researchers proved that a more extensive lymphadenectomy (> 40 RLNs) improved survival of cervical cancer patients with tumors sized > 4 cm [9]. However, proof on some serious adverse events suggests that cervical cancer patients with lymphadenectomy are more likely to undergo surgery-related systemic morbidity [13], and a more thorough lymphadenectomy could lead to postoperative complications and damage the immune system, such as lymphedema of the legs [14, 15]. Consequently, determining appropriate surgical scope could be conducive to avoid postoperative complications and improved survival of cervical cancer patients.

Traditional clinical variables, including tumor size, parametrial involvement, and so on, play important roles in the patient prognosis and FIGO staging system [16]. Nevertheless, conventional pathological variables are not reliable enough for finding optimal treatment strategies and predicting clinical prognosis. In terms of progression-free and overall survival, patients with similar pathological characteristics and clinical tumor stage have different prognosis. On account of the difficulty of predicting the prognosis of cervical cancer patients, it is urgent to find more novel clinical and prognostic factors for cervical cancer. Many factors, such as lymph node metastasis, initial symptom age, and days of catheter removal may provide effective information for appropriate therapeutic options and patient prognosis. In this study, we aimed to assess the independent prognostic value of clinical and potential prognostic factors in progression-free survival (PFS) in cervical cancer.

## Methods

### Study design and participants

A total of 100 cervical cancer patients of the Zhuhai Hospital of Traditional Chinese and Western Medicine were followed-up in this study between January 2017 and October 2019. Our daily diagnosis and treatment were in strict accordance with the guideline of the 2017 and 2019 NCCN. Totally, we included 100 cervical cancer patients, in whom 93 patients were cervical cancer of stage I-II, 6 patients were stage III, and the other one patient was cervical cancer stage IVa. According to the guideline, these seven patients were recommended to receive radiotherapy and chemotherapy, and were told that surgical treatment was not recommended. However, these patients insisted and strongly demanded surgical treatment, thus they voluntarily signed up for the surgery and was included in the follow-up plan. The exclusion criteria: according to the guidelines of the NCCN and clinical cervical cancer criteria, patients with previous other tumors receiving chemotherapy as well as radiotherapy and with surgical contraindications that cannot receive surgery were excluded from this study. Among the 100 patients, 7 cases were diagnosed with precancerosis after surgery and one case with relapse were also excluded from following analysis. Finally, there was 92 patients included in the following analysis. Lymph node metastases were diagnosed by pathological examination after lymphadenectomy. This study was approved by the institutional ethics committee and informed consent was obtained from all cervical cancer patients. Follow-up duration was from the date of primary therapy to the date of death or last contact (October 2019).

### Surgical procedures and pathological review

All the patients included in this study received surgical treatment for the first time. Among them, based on the NCCN guidelines and patients’ own decision, 5 patients underwent laparoscopic extensive total hysterectomy and bilateral salpingectomy (3 of stage Ia1, 1 of stage IVa, and 1 of stage IIIb) and other 87 patients underwent laparoscopic radical hysterectomy, pelvic lymph node dissection and partial para-aortic lymph node dissection. Twenty-four patients were submitted to bilateral salpingo-oophorectomy (4 of stage Ia1, 14 of stage Ib1, 3 of stage Ib2, 1 of stage IIb1, 1 of stage IVa, and 1 of stage IIIa). The extent of para-aortic lymph node dissection (PALND) transformed from para-aortic lymph node sampling to thorough lymphadenectomy up to the level of the renal vein. Pelvic lymph node dissection (PLND) included dissection of the internal iliac, external iliac, common iliac, and obturator lymph nodes on both sides. Parametrial lymph nodes were removed along with the parametrium. Para-aortic nodes and pelvic lymph nodes were both included in the retrieved lymph nodes count. Surgical techniques will improve over time. At the time of 2017, NCCN guidelines recommend radical total hysterectomy plus bilateral pelvic lymphadenectomy as the preferred surgical option and sentinel lymph node biopsy as an alternative method. Nowadays, sentinel lymph node biopsy is the first choice of surgical method for the patients with tumor less than 2 cm according to the NCCN guidelines [17].

### Statistical analysis

Kaplan–Meier method was used to assess the progression-free survival (PFS), and the log-rank test was used to analyze prognostic variables. Receiver operating characteristic (ROC) curve analysis was established to evaluate the prognostic performance of variables. *P*-value < 0.05 was considered statistically significant. All statistical data were analyzed by using SPSS Statistics 22.0 (SPSS, Chicago, IL, United States).

## Results

### Patients’ characteristics

Our study was based on data obtained from 92 cervical cancer patients between January 2017 and October 2019. The clinicopathological characteristics of 92 cervical cancer patients are shown in Table 1. Initial symptom age was categorized according to three roughly equal strata: 25–39 years (18 patients, 20.5%), and 40–75 years (70 patients, 79.5%). Regarding the FIGO stage (2018), stage I was the most common with 58 cases (63.0%), and 24 cases were stage II (26.1%), and 10 cases were stage III and stage IV (10.9%). The pathological classification was identified as follows: squamous cell carcinoma (63 cases, 68.5%), adenocarcinoma (15 cases, 16.3%), and others (14 cases, 15.2%). The mean age of cervical cancer patients was 49.6 years (range: 25–75 years), and the mean body mass index (BMI) was 23.1 kg/m2 (range: 17.2–35.4 kg/m2). The mean follow-up period was 14 months (range: 2–31 months). The mean number of lymph nodes dissected was 58 (range: 16–105) during surgery. After the operation, 65 patients (70.6%) received adjuvant radiotherapy or chemotherapy, whereas no further treatment was initiated in 27 patients (29.4%).

**Table 1:** Clinicopathological characteristics of the 92 cervical cancer patients included in this study

Characteristic	Patients	Disease Progression^#^	
		Yes	No
Pathological classification			
Squamous cell carcinoma	63 (68.5%)	4	59
Adenocarcinoma	15 (16.3%)	1	14
Others	14 (15.2%)	1	13
Initial symptom age^*^			
25–39	18 (20.5%)	3	15
40–75	70 (79.5%)	3	67
Days of catheter removal^**^			
2–7	8 (9.2%)	2	6
> 7	79 (90.8%)	3	76
Tumor stage			
Stage I	58 (63.0%)	1	57
Stage II	24 (26.1%)	2	22
StageIII + IV	10 (10.9%)	3	7
Adjuvant radiotherapy or chemotherapy			
Yes	65 (70.6%)	6	59
No	27 (29.4%)	0	27
BMI			
Normal	56 (60.9%)	6	50
Overweight+Obesity+Underweight	36 (39.1%)	0	36
Obstetrical history			
0–2	68 (73.9%)	4	64
> 2	24 (26.1%)	2	22
Retrieved lymph nodes count^***^			
16–30	9 (10.3%)	1	8
31–85	71 (81.6%)	2	69
86–105	7 (8.1%)	2	5
Uterine manipulator			
Yes	78 (84.8%)	5	73
No	14 (15.2%)	1	13
Pathological differentiation degree^****^			
Low differentiation	19 (25.3%)	2	17
Median differentiation	49 (65.3%)	4	45
High differentiation	7 (9.4%)	0	7
-	75 (81.5%)	5	70
+	17 (18.5%)	1	16

^*^Missing value *N* = 4; ^**^Missing value *N* = 5; ^***^Missing value *N* = 5; ^****^Missing value *N* = 17. ^#^Disease Progression: disease recurrence or death. *Abbreviations*: *BMI* body mass index, *HPV* human papillomavirus

### Prognostic significance of factors

**Fig. 1:**
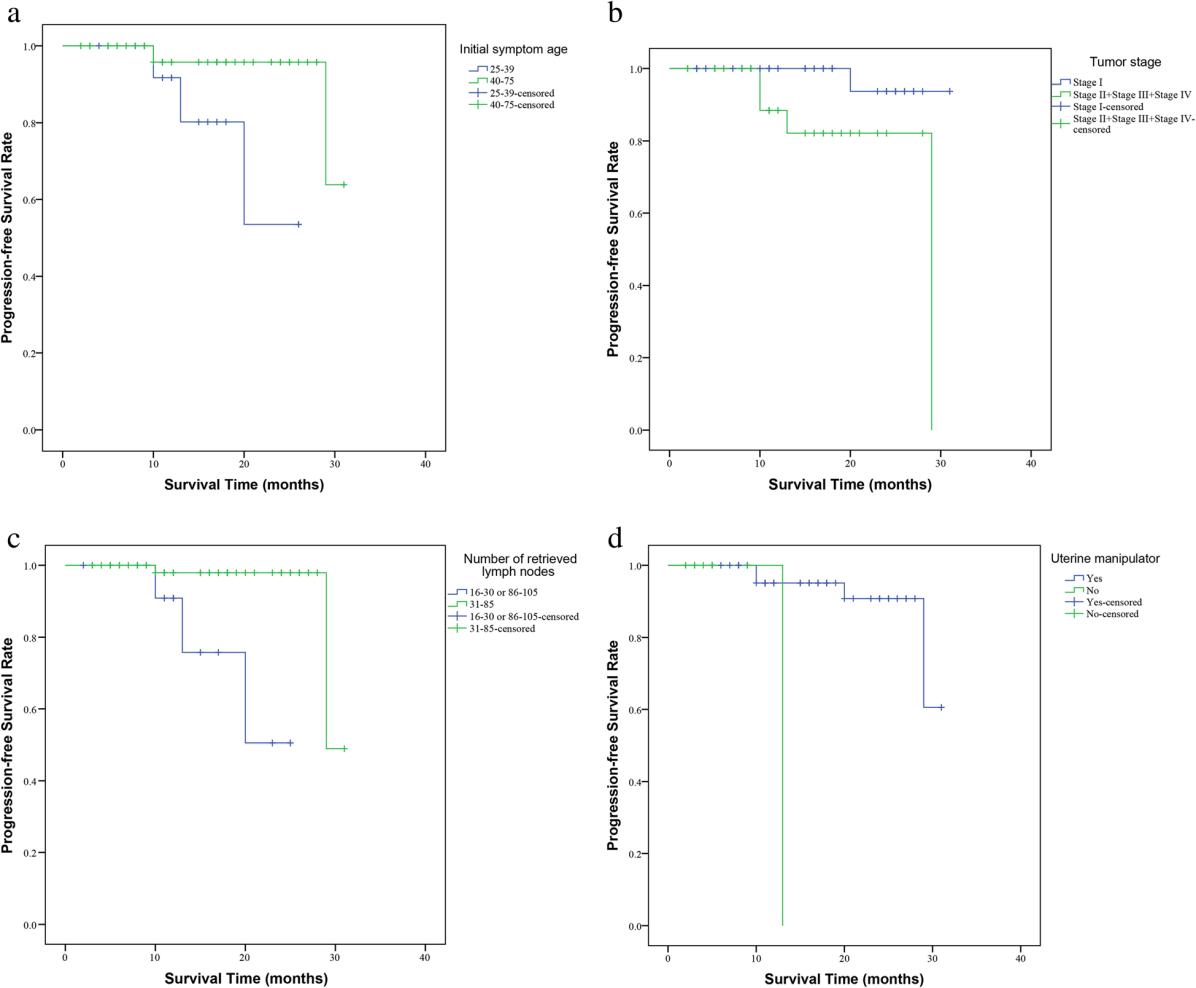
Kaplan-Meier curves of PFS in patients with cervical cancer. (**a**): Initial symptom age; (**b**): Tumor stage; (**c**): Retrieved lymph nodes count; (**d**): Uterine manipulator. PFS, progression-free survival

By generating Kaplan-Meier curves of progression-free survival (PFS), we found that the initial symptom age 25–39 group had significantly worse PFS (*P* = 0.020) compared to the initial symptom age 40–75 group (Fig. 1 a). Consistent with common sense, our results showed that the stage II, stage III and stage IV group had significantly worse PFS (*P* = 0.015) compared to the stage I (Fig. 1 b). Moreover, the retrieved lymph nodes count 31–85 group showed significantly better PFS (*P* = 0.002) compared to the retrieved lymph nodes count 16–30 or 85–105 groups (Fig. 1 c). And the using uterine manipulator group also showed significantly better PFS (*P* < 0.001) compared to the group without uterine manipulator (Fig. 1 d). As presented in Fig. S1, Kaplan-Meier curves of progression-free survival (PFS) suggested that BMI, post-operative bladder catheterization period, human papillomavirus (HPV), pathological classification, pathological differentiation degree, adjuvant radiotherapy or chemotherapy, obstetrical history, Ki-67, estrogen receptor (ER), progesterone receptor (PR), and P53 were not significant predictors for PFS (*P* > 0.05 for all) in cervical cancer patients.

### Correlation between post-operative bladder catheterization period and potential prognostic factors in cervical cancer

Bladder dysfunction is one of the most usual long-term side effects and complications after radical hysterectomy with the incidence of 8–80%. Our study tried to explore the association between the post-operative bladder catheterization period and potential prognostic factors in cervical cancer by Kruskal-Wallis test. As shown in Table 2, stage II, stage III and stage IV group had a significantly longer post-operative bladder catheterization period (*P* = 0.026) compared to the stage I group. In our study, we also found that the squamous cell carcinoma group had significantly longer post-operative bladder catheterization period (*P* = 0.023) compared to the adenocarcinoma and others group. Particularly, the post-operative bladder catheterization period showed a statistical difference between retrieved lymph nodes count 16–30, 31–85, and 86–105 group (*P* = 0.013). However, in the comparison of the characteristics of initial symptom age, BMI, HPV, uterine manipulator, obstetrical history and pathological differentiation degree, no significant difference in post-operative bladder catheterization period was observed between the groups (*P* > 0.05 for all).

**Table 2:** The association between the post-operative bladder catheterization period and potential prognostic factors

Characteristic	*N*	Mean	*P*-value
Tumor stage			
Stage I	55	39.39	0.026
Stage II + III + IV	32	51.92	
Retrieved lymph nodes count			
16–30	9	20.44	0.013
31–85	71	43.29	
86–105	7	51.71	
Pathological classification*			
Squamous cell carcinoma	59	48.34	0.023
Adenocarcinoma	14	36.21	
Others	13	29.38	
Obstetrical history			
0–2	63	43.09	0.585
> 2	24	46.40	
Initial symptom age**			
25–39	17	41.71	0.955
40–75	66	42.08	
BMI			
Normal	53	45.92	0.374
Overweight+Obesity+Underweight	34	41.00	
Pathological differentiation degree***			
Low differentiation	18	38.06	0.803
Median differentiation	46	35.79	
High differentiation	7	32.07	
HPV			
-	71	44.56	0.665
+	16	41.53	
Uterine manipulator			
Yes	73	43.32	0.567
No	14	47.54	

*Missing value *N* = 1; **Missing value *N* = 4; ***Missing value *N* = 16. *Abbreviations*: *BMI* body mass index, *HPV* human papillomavirus

### Combining the FIGO staging system and retrieved lymph nodes count to provide additional stratification

As shown in Fig. 1 c, the number of retrieved lymph nodes count was recommended as an independent prognostic factor. To investigate thoroughly cervical cancer patients with different oncological outcomes and find out more accurate indicators for cervical cancer, this study was implemented to investigate the prognostic roles of combining retrieved lymph nodes count and FIGO staging system in cervical cancer patients. Herein, cervical cancer patients were stratified by retrieved lymph nodes count and the FIGO staging system as follows: Group1, stage I disease with retrieved lymph nodes count 31–85; Group 2, stage I disease with retrieved lymph nodes count 16–30 or 86–105; Group 3, stage II+ III+ IV disease with retrieved lymph nodes count 31–85; Group 4, stage II+ III+ IV disease with retrieved lymph nodes count 16–30 or 86–105.

**Fig. 2:**
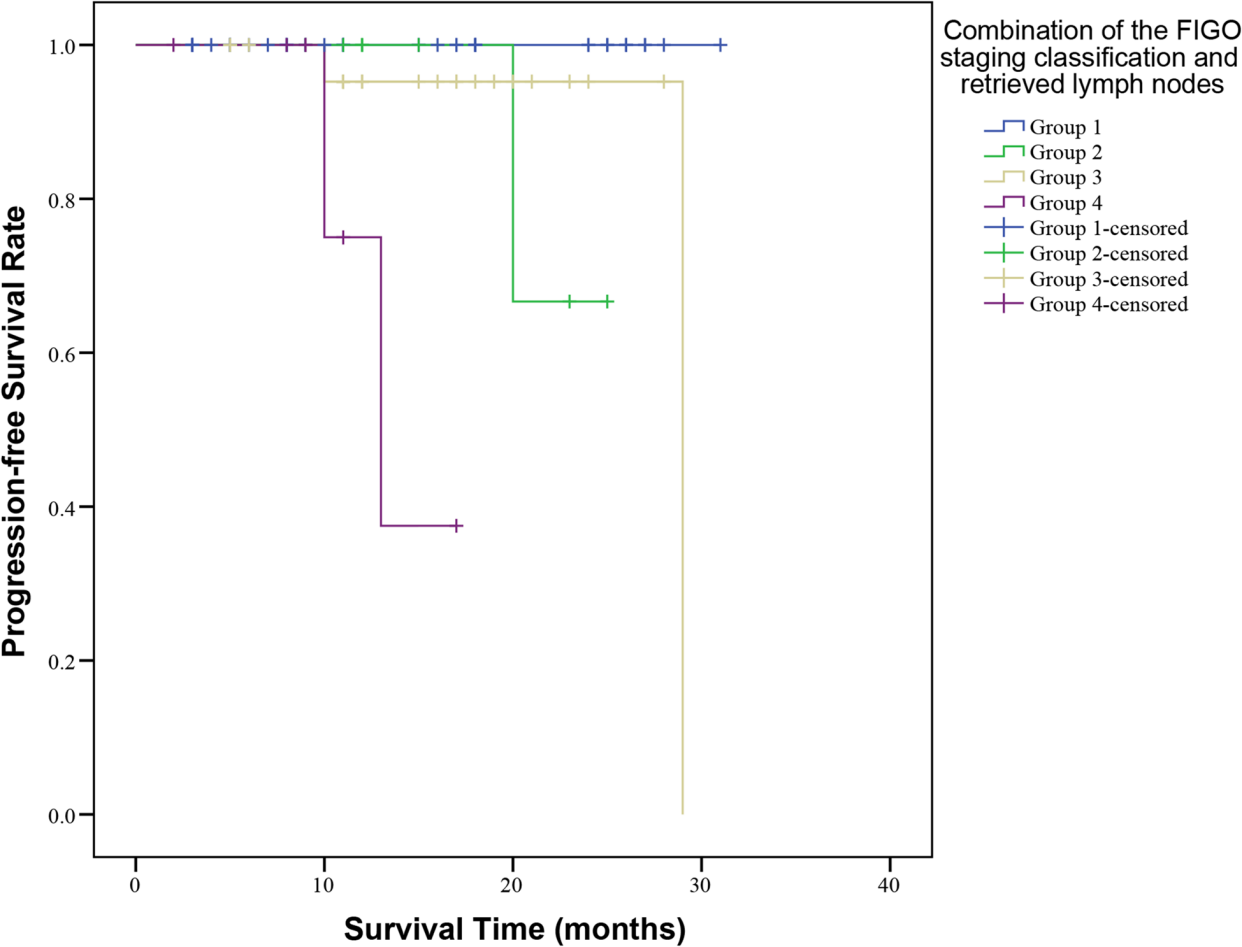
Kaplan-Meier curves of PFS related to the combination of retrieved lymph nodes count and FIGO stage of cervical cancer patients. PFS, progression-free survival. FIGO, International Federation of Gynecology and Obstetrics

**Fig. 3:**
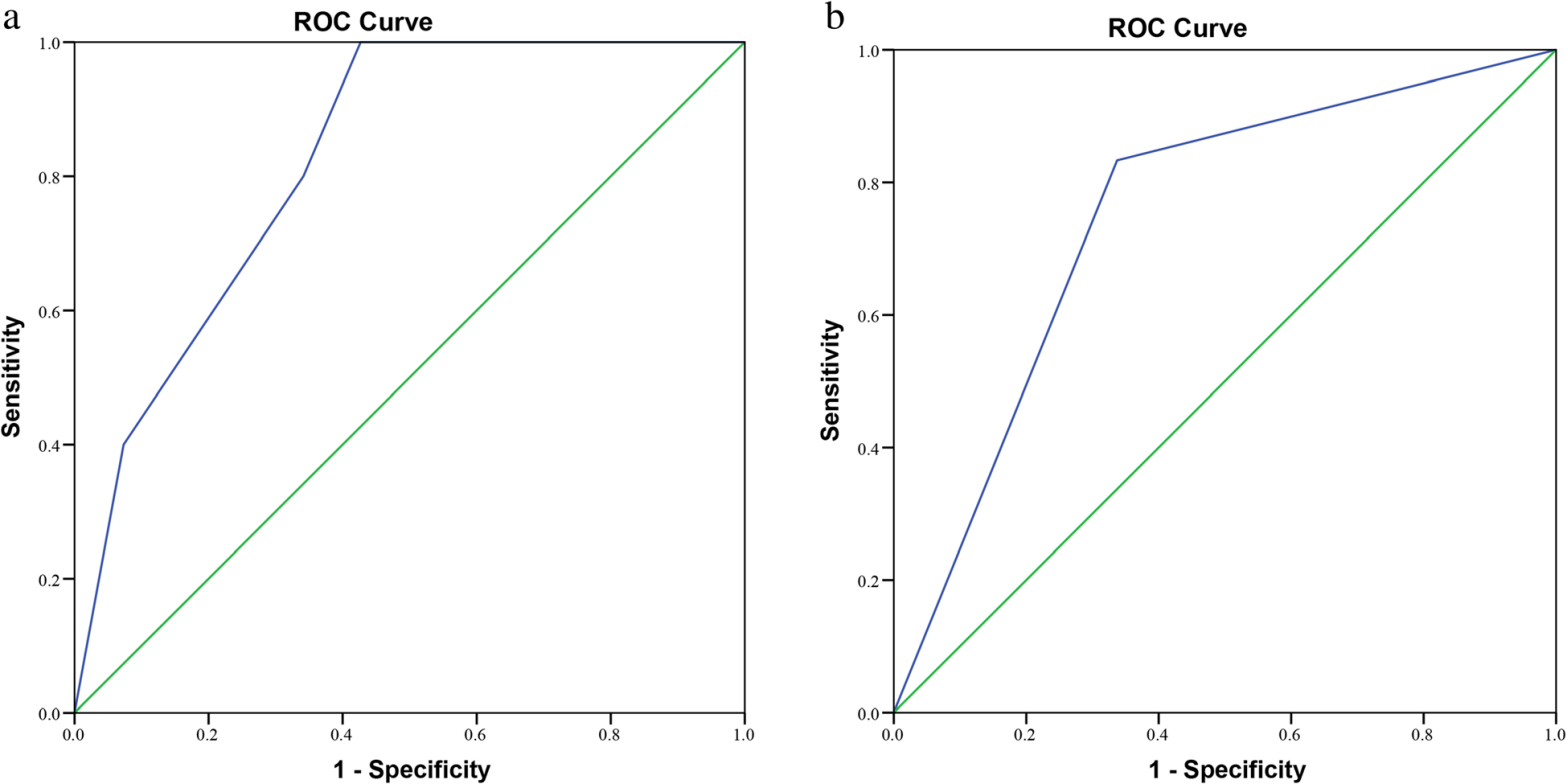
ROC curves of retrieved lymph nodes count and FIGO staging classification. **a**: Combination of retrieved lymph nodes count and FIGO staging classification; **b**: FIGO staging classification. ROC, receiver operating characteristic. FIGO, International Federation of Gynecology and Obstetrics

The log-rank test was used to compare the difference in survival between four groups, while the Kaplan-Meier method was used to draw a survival curve. The results revealed that Group 1 had significantly superior survival to the other groups (*P* < 0.001), and the Kaplan-Meier plot also showed that Group 1 had higher survival rates than the other groups (Fig. 2). Receiver operating characteristic (ROC) curve analysis was established to evaluate the prognostic prediction of the combination of retrieved lymph nodes count (31–85 and 16–30 or 86–105) and FIGO staging classification (stage I, II, III and IV). As shown in Fig. 3, the AUC of retrieved lymph nodes count combined with FIGO stage (AUC = 0.826, 95% CI: 0.689–0.962) was better than FIGO staging alone (AUC = 0.748, 95% CI: 0.561–0.935) in prognostic prediction for cervical cancer patients. These results indicated that the combination of retrieved lymph nodes count (31–85 and 16–30 or 86–105) and FIGO staging classification (stage I, II, III and IV) could act as a potential predictor for prognosis of cervical cancer patients.

## Discussion

Many gynecologic oncologists are dubious about the scope of lymphadenectomy as treating cervical cancer patients with radical hysterectomy. Our study evaluated the effect of the extent of lymphadenectomy on the survival of cervical cancer patients. According to the suggestion of the Gynecologic Oncology Group, the retrieved lymph nodes count that could define an adequate lymphadenectomy was 10 or more [18]. It should maintain a minimum of one from each hemi-para-aortic lymphadenectomy and four lymph nodes from each hemipelvic lymphadenectomy. However, an appropriate number of retrieved lymph nodes for cervical cancer remains questionable. Categorization of cervical cancer patients in published studies is diverse. There is evidence investigated by other researchers demonstrated that divided cervical cancer patients into two groups based on a retrieved lymph nodes cut point of 40, which the patients could be separated almost equally [9]. Pieterse et al. divided cervical cancer patients into two groups based on the retrieved lymph nodes, a median number of 18 [12]. In our current study, number of retrieved lymph nodes had a statistically significant effect on PFS of cervical cancer patients. Findings of our current study demonstrate that patients with retrieved lymph nodes count 31–85 showed better prognosis than patients with retrieved lymph nodes count 16–30 or 86–105 group. This finding may be conducive to determine surgical scope before surgery for cervical cancer patients. From the results, we can find lymph nodes are not always better dissected as much as possible. We already know the tumors also spread along the lymphatic system. We hypothesis if a higher number of retrieved lymph nodes in a similar dissection area of patients could indicate a more developed lymphatic reflux system, the patients with a more developed lymphatic system would have a higher risk of tumor cell metastasis. Therefore, patients of retrieved lymph nodes count more than 85 in this study had worsening prognosis. This finding may be conducive to determine surgical scope before surgery for cervical cancer patients, and further clinical studies and more data analysis are needed to confirm the details.

Published researches related to the possible significance of initial symptom age of cervical cancer patient as a prognostic factor are contradictory. In several studies, the prognosis is significantly worse in younger cervical cancer patients [19, 20]. However, in other studies, there is no difference in the survival rates among cervical cancer patients in different age groups [21, 22]. Our results showed that the initial symptom age 25–40 group had significantly worse PFS (*P* = 0.020), and the initial symptom age may be prognostic factor. The relational factors in this result are still unclear. It could not be explained by HPV infection respectively, disease pattern and aggressive characteristics were also potential factor [23]. Agreed with other research, our current study corroborated that stage I cervical cancer patients had significantly better PFS (*P* = 0.015) compared to the stage II, stage III and stage IV cervical cancer patients [24].

While being widely adopted, the using of uterine manipulator in laparoscopic treatment represents a controversial issue. Recent studies have demonstrated that laparoscopic surgery was correlated with worse PFS and shorter overall survival than open abdominal approach among cervical cancer patients [25, 26]. Uterine manipulator was hypothesized as one of suspected causes responsible for the bad prognosis. However, other studies showed that the use of uterine manipulator in laparoscopic approach does not related to the risk of recurrence and worse PFS [27]. In our current study, the using of uterine manipulator had a statistically significant effect on PFS of cervical cancer patients (*P* < 0.001). Findings of our current study demonstrated that the using of uterine manipulator in laparoscopic treatment showed a better prognosis than patients without uterine manipulator using group. In surgical process of this study, the manipulator was carefully treated and its movements were minimized after placement. The direction should be maintained. The force was controlled as much as possible during the whole surgical process to avoid its violent extrusion of the tumor, which would cause the metastasis of cancer cells. After our observation, if these manipulations above could be achieved, the using of the manipulator could help the surgeon better judging the size of the tumor and surrounding tissue conditions to help patients get better PFS.

A novel discovery of our study was to verify the prognostic values of retrieved lymph nodes count combining with the FIGO staging system, which had never been investigated in cervical cancer before. Our study separated cervical cancer patients into four groups according to the retrieved lymph nodes count and the FIGO staging system. Kaplan-Meier survival curve analysis showed that Group 1(stage I disease with retrieved lymph nodes count 31–85) had higher survival rates than the other groups. Receiver operating characteristic (ROC) curve analysis also showed the retrieved lymph nodes count combined with the FIGO stage was better than FIGO staging alone in prognostic prediction for cervical cancer patients. In summary, our study indicates that retrieved lymph nodes count combined with the FIGO stage could be a potential prognostic factor for cervical cancer patients. The combining of retrieved lymph nodes count and FIGO staging system prognostic system would provide added risk stratification, so cervical cancer patients with predicted worse prognosis should receive more aggressive therapeutic regimens.

To date, there is no reported evidence that the post-operative bladder catheterization period has a direct impact on survival. Intraoperative injury of pelvic floor nerve, ureter and bladder were acknowledged factors that affected the days of catheter removal. The time of catheter removal is related to the scope of the operation. Retrieved lymph nodes count reflects the range of surgical dissection, which was found as prognostic factor in this study. Therefore, this study analyzes correlations between potential prognostic factor and days of catheter removal. As shown in results, in the comparison of tumor stage, retrieved lymph nodes count, and pathological type, significant difference in post-operative bladder catheterization period was observed between the groups (*P* < 0.05 for all). Based on these results, we raised the possibility that the post-operative bladder catheterization period could be potential indirect acting factor for prognosis of cervical cancer patients. Although it is difficult to demonstrate this hypothesis at present study, further relevant research and verification in the future were expected.

There is no crucial evidence regarding the impact of the pathological differentiation degree of cervical cancer on PFS. Some researches indicate that poorly-differentiated squamous cell cervical carcinoma has a worse prognosis [28], whereas other researches did not identify [29, 30]. Another controversial prognostic factor in patients with cervical cancer is the pathological classification [31]. While some studies reported that patients with uterine cervix adenocarcinoma have unfavorable outcomes [32, 33], others identified no significant differences between cervical adenocarcinoma and squamous cell carcinoma [24, 34]. In our current study, it was failed to show pathological differentiation degree and pathological classification were independent prognostic factors. Moreover, our study included four cervical patients with lymphatic obstruction. We speculated that it might connect with retrieved lymph nodes, the scope of lymphadenectomy, and the surgical method. Because the number of samples was limited, our research had some limits. A larger number of cervical cancer samples need to be collected and more studies are also needed to corroborate our speculation.

The standard treatment for recurrence cervical cancer is controversial. There are three diverse treatment choices for local recurrence or persistent cancer: systemic therapy or best supportive care or pelvic exenteration [17]. Several studies have shown that diverse surgical approach used in recurrence or persistent cancer could influence the prognosis of cervical cancers patients. Vaginectomy appears to a salvage treatment, for instance, exenteration or radiotherapy with a bottommost impact on quality of life in appropriately high-risk patients [35]. Lateral endopelvic resection (LEPR) was defined as en bloc resection of a pelvic tumor with pelvic side wall (PSW) structures including sidewall muscle, or bone, or major vascular structure, or major nerve to attain negative pathologic margins [36]. It is a curative potential therapeutic option for persistent or recurrent malignancy infiltrating the PSW, especially if chemoradiation failed, associated with prolonged survival [37].

Our current study has several limitations. Firstly, as a retrospective study, the bias could not be neglected. Although 92 cervical cancer patients were included in this study, it is possible that the results might be affected by some confounding factors. Secondly, our study was built on information from a single healthcare institution of a finite geographic region. Therefore, further confirmatory studies at other institutions should be carried out in the future. Finally, the study is still being followed-up and we will continue to follow up the patients in this study until death.

## Conclusions

In this study, we assessed the independent prognostic value of clinical and potential prognostic factors in progression-free survival (PFS) in cervical cancer. Number of retrieved lymph nodes, initial symptoms age, uterine manipulator, and retrieved lymph nodes count combining with FIGO staging system were identified as potential prognostic factors for cervical cancer patients.

## Supplementary Information

**Additional file 1: Figure S1.** Kaplan-Meier curves of PFS in patients with cervical cancer. (a): Number of births; (b): Adjuvant radiotherapy or chemotherapy; (c): P53; (d): Ki-67; (e): ER; (f): PR; (g): BMI; (h): HPV; (i): Pathological type; (j): Pathological differentiation degree. PFS, progression-free survival.

## Data Availability

The datasets used and/or analyzed during the current study available from the corresponding author on reasonable request.

## Abbreviations

PFS: Progression-free survival
ROC: Receiver operating characteristic
LMICs: Low and middle-income countries
NCCRC: National Central Cancer Registry of China
FIGO: International Federation of Gynecology and Obstetrics
RLN: Retrieved lymph nodes
PALND: Para-aortic lymph node dissection
PLND: Pelvic lymph node dissection
HPV: Human papillomavirus
ER: Estrogen receptor
PR: Progesterone receptor
