# Supplementary material for: Potential prognostic factors in progression-free survival for patients with cervical cancer
Source: BMC Cancer. 2021 May 10;21:531. doi: 10.1186/s12885-021-08243-3 (PMC8112015; doi:10.1186/s12885-021-08243-3)
Supplement: Supplementary file 1 — Additional file 1: Figure S1. Kaplan-Meier curves of PFS in patients with cervical cancer. (a): Number of births; (b): Adjuvant radiotherapy or chemotherapy; (c): P53; (d): Ki-67; (e): ER; (f): PR; (g): BMI; (h): HPV; (i): Pathological type; (j): Pathological differentiation degree. PFS, progression-free survival. [file 12885_2021_8243_MOESM1_ESM.pdf]

Potential prognostic factors in progression-free survival for patients with cervical cancer

Hui-Hui Chen[1#], Wei-Yu Meng[2#], Run-Ze Li[2#], Qing-Yi Wang[1], Yu-Wei Wang[2], Hu-Dan Pan[2], Pei-Yu Yan[2], Qi-Biao Wu[2], Liang Liu[2], Xiao-Jun Yao[2*], Min Kang[1*] & Elaine Lai-Han Leung[1,2*]

1 Zhuhai Hospital of Traditional Chinese and Western Medicine, Zhuhai City, Guangdong, P.R. China

2 State Key Laboratory of Quality Research in Chinese Medicine/Macau Institute for Applied Research in Medicine and Health, Macau University of Science and Technology, Macau (SAR), China

**Correspondence: xjyao@must.edu.mo (X.J.Y); lhleung@must.edu.mo (E.L.-H.L.); Tel.: +853-8897-2409 (E.L.-H.L.); Fax: +853-2882-2799*

#These authors contribute equally.

SUPPLEMENTARY INFORMATION – Content list

Materials and methods

Study design and participants

Cervical cancer screening is carried out according to the international three stages in China. In this study, 12 asymptomatic patients were all diagnosed by routine cervical cancer screening.

In our hospital, all patients underwent routine blood routine, biochemistry, coagulation, liver function test, SCC, CEA, AFP, CA125, electrocardiogram, chest CT plain scan with enhancement, pelvic MR plain scan with enhancement and other examinations before surgery. Due to the high cost and false negatives of PET and CT, such tests are only added when significant lymph node enlargement occurs on routine imaging. In this study, most patients of the pelvic lymph nodes were negative before surgery, and a few had slightly enlarged lymph nodes.

FIGURE

- Figure S1 - Kaplan-Meier curves of PFS in patients with cervical cancer. (a): Number of births; (b): Adjuvant radiotherapy or chemotherapy; (c): P53; (d): Ki-67; (e): ER; (f): PR; (g): BMI; (h): HPV; (i): Pathological type; (j): Pathological differentiation degree

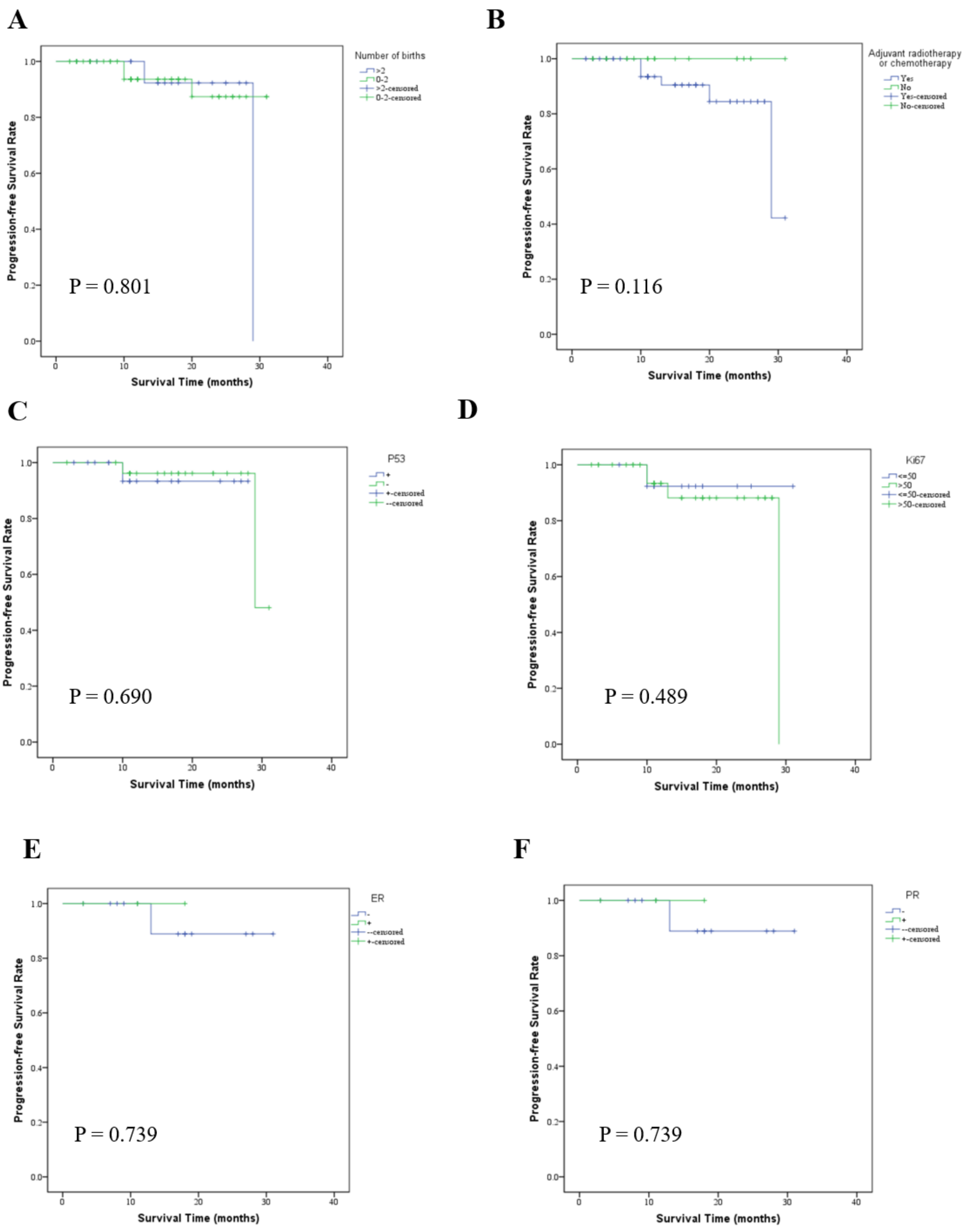
A
Number of births
>2
0-2
>2-censored
0-2-censored
Progression-free Survival Rate
Survival Time (months)
P = 0.801
B
Adjuvant radiotherapy or chemotherapy
Yes
No
Yes-censored
No-censored
Progression-free Survival Rate
Survival Time (months)
P = 0.116
C
P53
+
-
+-censored
--censored
Progression-free Survival Rate
Survival Time (months)
P = 0.690
D
Ki67
<=50
>50
<=50-censored
>50-censored
Progression-free Survival Rate
Survival Time (months)
P = 0.489
E
ER
-
+
--censored
+-censored
Progression-free Survival Rate
Survival Time (months)
P = 0.739
F
PR
-
+
--censored
+-censored
Progression-free Survival Rate
Survival Time (months)
P = 0.739

G

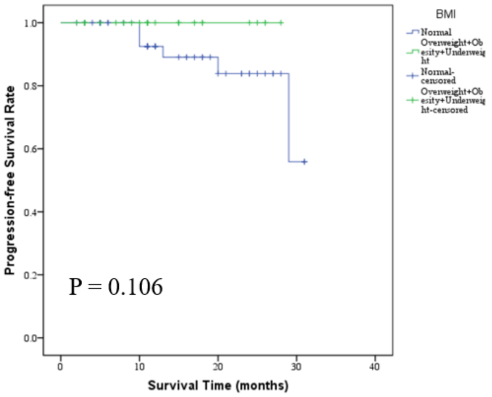
BMI
Normal
Overweight+Ob
esity+Underweig
ht
Normal-
censored
Overweight+Ob
esity+Underweig
ht-censored
Progression-free Survival Rate
Survival Time (months)
P = 0.106

H

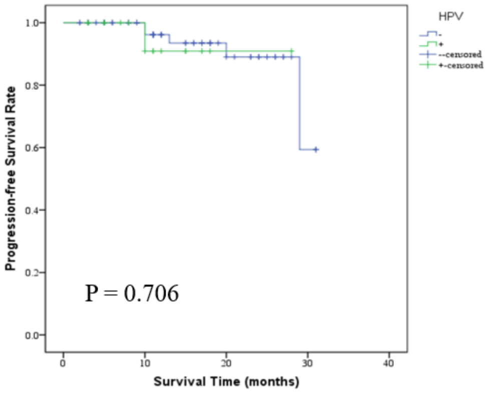
HPV
-
+
--censored
+-censored
Progression-free Survival Rate
Survival Time (months)
P = 0.706

I

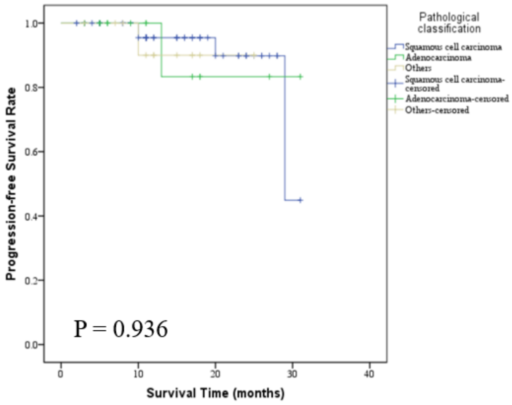
Pathological
classification
Squamous cell carcinoma
Adenocarcinoma
Others
Squamous cell carcinoma-
censored
Adenocarcinoma-censored
Others-censored
Progression-free Survival Rate
Survival Time (months)
P = 0.936

J

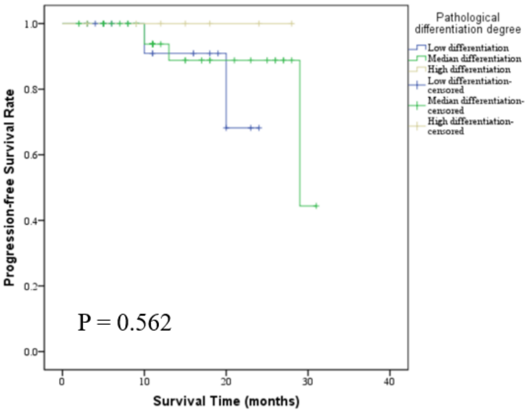
Pathological
differentiation degree
Low differentiation
Median differentiation
High differentiation
Low differentiation-
censored
Median differentiation-
censored
High differentiation-
censored
Progression-free Survival Rate
Survival Time (months)
P = 0.562

K

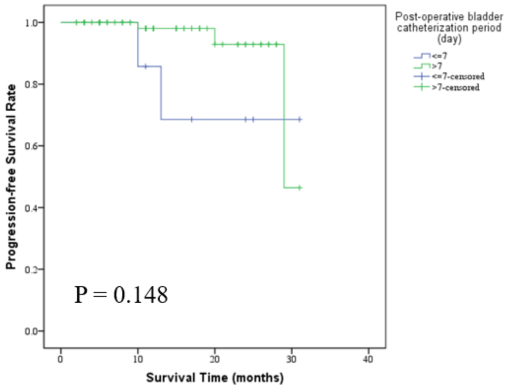
Post-operative bladder
catheterization period
(day)
<=7
>7
<=7-censored
>7-censored
Progression-free Survival Rate
Survival Time (months)
P = 0.148
